# Irisin targets the HK1–glycolysis–NLRP3 pyroptosis axis to prevent chronic kidney disease-associated vascular calcification

**DOI:** 10.1080/0886022X.2025.2610545

**Published:** 2026-01-07

**Authors:** Weijia Xu, Peiwen Wang, Xiaowen Zhu, Zongli Zhang, Xingtong Dong, Ran An, Qi Pang, Aihua Zhang

**Affiliations:** ^a^ Department of Nephrology, Xuanwu Hospital, Capital Medical University, Beijing, P. R. China; ^b^ Department of Nephrology, Taihe Hospital, Hubei University of Medicine, Shiyan, Hubei, P. R. China; ^c^ Department of Gastrology, Taihe Hospital, Hubei University of Medicine, Shiyan, Hubei, P. R. China; ^d^ Institute of Pediatric Disease, Taihe Hospital, Hubei University of Medicine, Shiyan, Hubei, P. R. China; ^e^ Clinical Research Center for Umbilical Cord Blood Hematopoietic Stem Cells, Taihe Hospital, Hubei University of Medicine, Shiyan, Hubei, P. R. China

**Keywords:** Irisin/Fndc5, Hexokinase1, glycolysis, pyroptosis, vascular calcification, chronic kidney disease

## Abstract

Vascular calcification (VC) in chronic kidney disease (CKD) remains a critical clinical challenge with limited therapeutic options. This study investigates the regulatory role of HK1-mediated glycolysis in NLRP3 inflammasome-driven pyroptosis during chronic kidney disease (CKD)-associated vascular calcification (VC) and the protective effects of Irisin/FNDC5. Using β-glycerophosphate-treated vascular smooth muscle cells (VSMCs) and CKD mouse models, we employed transcriptomic analysis, FNDC5 genetic knockout, and overexpression strategies to dissect HK1’s mechanistic contribution. Results revealed that HK1-dependent glycolysis amplifies NLRP3 inflammasome activation and pyroptosis in calcifying VSMCs, while Irisin suppressed HK1 expression and glycolytic flux, thereby inhibiting NLRP3-driven pyroptosis. Irisin precursor Fndc5 knockout exacerbated aortic calcification and upregulated HK1-NLRP3 signaling in CKD mice, whereas Irisin administration reversed these effects. This study demonstrates that HK1-driven glycolysis acts as a metabolic driver of NLRP3-mediated pyroptosis in CKD-associated vascular calcification. Irisin suppresses this HK1–glycolysis–inflammasome axis, thereby attenuating vascular smooth muscle cell pyroptosis and calcification. These findings reveal a previously unrecognized HK1–glycolysis–NLRP3 pathway as a metabolic-inflammatory target and suggest that Irisin/HK1 modulation may represent a novel therapeutic strategy for CKD-related vascular calcification.

## GRAPHICAL ABSTRACT

**Figure:**
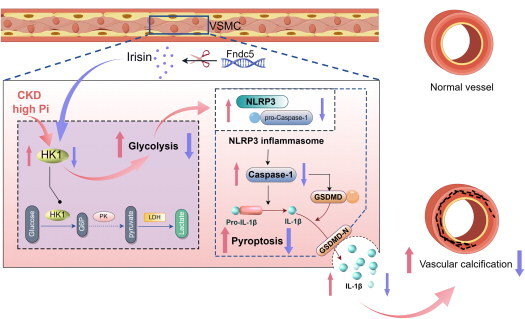

## 1. Introduction

Vascular calcification (VC), a serious complication of chronic kidney disease, is a predictor of mortality in CKD patients. Numerous studies have revealed that medial calcification is predominant in CKD-associated VC [1]. As vascular smooth muscle cells (VSMCs) are the dominant components of the arterial tunica media, morphologic and functional changes in VSMCs determine the degree of medial calcification. However, the mechanism of vascular medial calcification in CKD patients remains unclear, and no agent that can effectively prevent VC is currently available.

Accumulating evidence has revealed that energy metabolism is involved in cardiovascular diseases [2,3]. Among all energy metabolism pathways, glucose metabolism is the major contributor to the pathogenesis of cardiovascular disease [4,5]. Under physiological conditions, oxidative phosphorylation is capable of maintaining the normal phenotype and function of VSMCs [6,7]; however, under exposure to calcifying environments, such as high-fat or high-glucose conditions, VSMCs exhibit glycolysis addiction and rely on glycolysis as their main source of energy [8,9]. Glycolysis is an oxygen-independent metabolic pathway that converts glucose to pyruvate and then produces lactic acid. However, under certain circumstances, cells can rely primarily on glycolysis rather than oxidative phosphorylation, even in the presence of oxygen, a phenomenon known as the Warburg effect. Glycolysis takes place in the cytosol and is catalyzed by hexokinase (HK), phosphofructokinase (PKF), and pyruvate kinase (PK), which carry out three key irreversible regulatory steps [10]. Recently, lactate, the end product of glycolysis, has been shown to upset the equilibrium of mitochondrial homeostasis in VSMCs, hence promoting VC [11]. Moreover, increased expression of several glycolytic enzymes has been observed in VSMCs under CKD conditions [2]. However, the contribution of glycolysis to CKD-associated VC and the possible mechanisms involved remain poorly understood.

Irisin is a 112-amino acid myokine that is generated by the cleavage of its precursor, fibronectin type III domain-containing protein 5 (Fndc5). It is considered a novel regulator of many physiological and pathological processes, such as energy homeostasis, adipose tissue browning, bone synthesis, and insulin resistance [12–14]. Previous studies have demonstrated that Irisin exerts protective effects against disease development by regulating the glycolytic process. Li et al. [15] reported that Irisin plays a protective role in acquired aplastic anemia by inhibiting the activation of mitochondrial fission proteins and enhancing aerobic glycolysis in hemopoietic stem cells. Zhang et al. [16] revealed that Irisin increased mitochondrial respiration and glycolysis in visceral adipocytes to promote anti-inflammatory activity in fat tissue. Another report showed that Irisin preferentially stimulated aerobic glycolysis to promote the proliferation and differentiation of osteoblast lineage cells during bone formation [17]. In recent years, several studies have revealed that glycolysis is an important trigger that activates the NLRP3 inflammasome [18–20]. Our team previously reported that Irisin protects against CKD-associated VC by inhibiting NLRP3-dependent pyroptosis in VSMCs [21]. At present, glycolysis and pyroptosis play a very important role in VC under CKD context. However, it has not been reported whether high phosphate-induced glycolysis can activate VSMCs pyroptosis, and whether the protective effect of Irisin on VC occurs through the regulation of glycolysis to inhibit NLRP3 inflammasome activation and subsequent VSMCs pyroptosis.

In this study, we investigated the role of Irisin linking glycolysis and pyroptosis in VSMCs and CKD-associated VC by *in vitro* study and *in vivo* animal study. Our data will provide the novel perspectives on the role of Irisin/Fndc5 in CKD-associated VC.

## 2. Materials and methods

### 2.1. Animals and treatments

All animal care and experimental protocols complied with the National Institutes of Health (NIH) Guide for the Care and Use of Laboratory Animals. Ethics declarations for the mouse experiments were approved by the Institutional Animal Care and Use Committee (IACUC) of Capital Medical University. Male 6-week-old C57BL/6J mice (WT mice) were acquired from the National Model Animal Research Center of Nanjing University. Fndc5-KO C57BL/6J mice (Fndc5 KO mice) were purchased from Jiangsu Gem Pharmatech Biological Company (Nanjing, Jiangsu, China); the gene encoding the Irisin precursor Fndc5 was knocked out *via* CRISPR/Cas9 technology. The mice were housed in a controlled environment (temperature: 20–25 °C; 50 ± 5% humidity; and 12-h light–dark cycle) and had unrestricted access to sterile food and water. WT mice were randomly divided into three groups: the WT group, CKD group, and Irisin + CKD group. Fndc5 KO mice were randomly divided into two groups: the Fndc5 KO group and the Fndc5 KO CKD group. The WT group and Fndc5 KO group were provided a standard normal diet that contained 1.2% calcium and 0.6% phosphate throughout the entire process. The CKD model was induced by feeding mice a chow diet containing 0.25% adenine (A8626; Sigma-Aldrich, St. Louis, MO, USA) for 4–6 weeks, and the model establishment was confirmed by measuring blood urea nitrogen (BUN) and creatinine (Cr) levels. Medial calcification was induced by a high-calcium and high-phosphate diet (3% calcium and 1.8% phosphate) for 12–16 weeks on the basis of the CKD model, which was combined with the cutaneous administration of calcitriol (active vitamin D3; 1 μg/kg; twice a week; Sigma-Aldrich, St. Louis, MO, USA) [22]. For the Irisin + CKD group, purified Irisin (Phoenix Pharmaceuticals, Burlingame, CA, USA) was injected during the medial calcification induction period (2 µg in 100 µl of sterile normal saline per mouse *via* the tail vein, two times a week for 12–16 weeks).

All mice were euthanized by cervical dislocation under 2.5% isoflurane anesthesia *via* a vaporizer at the end of the experiment [23]. Aortas were isolated, collected, and either processed for Western blot analysis or embedded in paraffin for subsequent analyses. Blood samples were collected, and BUN, Cr, calcium, and phosphate levels were measured *via* microplate test kits (Nanjing Jiancheng Bioengineering Institute, Nanjing, China) following the manufacturer’s instructions.

### 2.2. Cell culture and treatments

Mouse aortic vascular smooth muscle cells (MOVAS-1) were purchased from Warner Bio Co., Ltd. (Wuhan, China) and incubated in DMEM (HyClone brand, Cytiva, Marlborough, MA, USA) supplemented with 10% fetal bovine serum (FBS, Gibco brand, Thermo Fisher Scientific, Waltham, MA, USA) and 1% penicillin/streptomycin (Sigma-Aldrich, St. Louis, MO, USA) in a 5% CO_2_ incubator at 37 °C. Experiments were performed using VSMCs between passages 6 and 10.

A Fndc5 knockout cell line was constructed *via* the use of lentiviral vectors encoding short hairpin RNAs (shRNAs) targeting mouse Fndc5 (GeneChem Company, Shanghai, China). Briefly, VSMCs were transfected with shRNA-Fndc5 lentivirus (shRNA-Fndc5) or an empty lentiviral vector (shRNA-NC) with polybrene (8 μg/ml, Sigma-Aldrich, St. Louis, MO, USA). The transfected cells were then subjected to selection with puromycin (3.0 μg/ml) for 4–5 days. Stable Fndc5 knockdown in cells was verified by Western blot.

For the glycerophosphate (β-GP) treatment group, VSMCs were maintained in culture medium containing β-GP (10 mM; G9422; Sigma-Aldrich, St. Louis, MO, USA) for the indicated times, as described in the figure legends. For the β-GP + Irisin cotreatment group, VSMCs were first pretreated in the culture medium with 100 ng/ml Irisin (8880-IR; R&D Systems, Minneapolis, MN, USA; dissolved in PBS), and then, 10 mM β-GP was added at the specified times. The medium was changed every 2–3 days.

### 2.3. Calcification assessment

Alizarin red staining was used to detect VSMCs calcification. Alizarin red powder (2.0 g; Sigma-Aldrich, St. Louis, MO, USA) was dissolved in 100 mL of ultrapure water, and the pH was adjusted to 4.2, generating a 2% Alizarin red solution. VSMCs were collected after the indicated treatments, rinsed in PBS 3 times, fixed for 10 min with 4% paraformaldehyde (PFA), and exposed to Alizarin red solution for 15–20 min in an incubator. The cells were then washed five times with distilled water and imaged under a light microscope (Nikon, DS-U3, Japan).

Von Kossa staining was used to identify mouse aorta calcification [24,25]. Paraffin sections of mouse aortas were deparaffinized, rehydrated, and immersed in a 1% silver nitrate solution under an ultraviolet lamp for 1–2 h, followed by treatment with 5% sodium thiosulfate for 5 min to remove any unreacted silver. Images were taken under a microscope (Nikon DS-U3, Japan). Calcium content measurements were performed to assess cell and tissue calcium concentrations. Lysates of VSMCs and mouse aortic tissues were prepared as previously described [25]. Calcium accumulation was quantified and normalized to the protein concentration of each culture. The calcium content was measured *via* quantitative colorimetric determination using a Quanti Chrom Calcium Assay Kit (C004-2-1, Nanjing Jiancheng Bioengineering Institute, Nanjing, China).

### 2.4. Western blot

Target proteins extracted from cultured cells and mouse aortic tissues were evaluated *via* Western blot analysis. Protein lysates were obtained with RIPA buffer (Beyotime, Shanghai, China, P0013) containing protease and phosphatase inhibitor cocktail. The lysates were subsequently sonicated, quantified, and boiled for 10 min. Equal amounts of protein were electrophoresed *via* SDS–PAGE before being transferred to polyvinylidene fluoride membranes (Millipore, St. Louis, MO, USA), after which the membranes were placed in 5% skim milk for 2 h at room temperature. The membranes were incubated at 4 °C overnight with the following primary antibodies: anti-HK1 (1:1000; Cell Signaling Technology, Danvers, MA, USA, 2024S), PKM (1:1000; Cell Signaling Technology, Danvers, MA, USA, 4053), anti-NLRP3 (1:500; Abcam, Waltham, MA, USA, ab270449), anti-GSDMD-N (1:500; Cell Signaling Technology, Danvers, MA, USA, 10137), anti-Caspase-1 (1:1000; Abcam, Waltham, MA, USA, ab138483), and anti-β-actin (1:5000; Proteintech, Wuhan, China, 14395-1-AP). The membranes were subsequently incubated with HRP-conjugated secondary antibodies (1:5000; Abcam, Waltham, MA, USA, ab205718) at 37 °C for 1 h. An enhanced ECL reagent (Thermo Fisher Scientific, Waltham, MA, USA, 34580) was used to visualize the blots, and ImageJ software was used to quantify the band density.

### 2.5. Quantitative real-time polymerase chain reaction (RT–qPCR)

RT–qPCR was used to assess the gene expression levels of HK1, HK2, PKM, and LDHA; β-actin was used as an internal control for normalization. Each sample was analyzed three times. Total RNA was extracted from each group with TRIzol reagent (Invitrogen, Waltham, MA, USA). The RNA was reverse transcribed into cDNA *via* a reverse transcription system (Takara, Kusatsu, Shiga, Japan, RR036A) before RT–qPCR analysis was performed. SYBR Green dye was used for RT–qPCR on a Biosystems 7500 Real-Time PCR System (Applied Biosystems, USA). The PCR primers used in this study were obtained from Genechem Company, and the sequences are provided in Supplementary Table 1.

### 2.6. Transfection assays

siRNAs targeting HK1 and HK1 overexpression plasmids (pcDNA 3.1- HK1) were constructed by Genechem Company（Shanghai,China）. Nontargeting negative control siRNA and empty pcDNA 3.1 plasmids were used as the negative controls. Both siRNA and DNA plasmid transfection were carried out *via* Lipofectamine 8000 (Beyotime, Shanghai, China, C0533). VSMCs were treated according to the experimental design 24 h after transfection, and a second transfection procedure was performed if the experiment lasted more than 3 days. Transfection efficiency was assessed by Western blot.

### 2.7. Glucose uptake and glycolytic activity assay

#### 2.7.1. Glucose uptake assay

The cells (5 × 10^4^ cells per well) were seeded in a 12-well plate for 24 h before indicated treatment. After stimulation, cells were equilibrated in glucose-free medium for 2 h prior to treatment with 100 μM 2-NBDG (Thermo Fisher Scientific, Waltham, MA, USA, N13195). After incubation for 60 min, the cells were collected and washed three times with PBS, and then observed *via* a fluorescence microscopy. During this process, 2-NBDG (2-N-(7-Nitrobenz-2-oxa-1,3-diazol-4-yl) amino-2-deoxy-d-glucose;), a glucose analog, was used as a tracer to evaluate the cells’ glucose uptake capacity. The fluorescence intensity at 488 nm was detected by a fluorescent microscope at 20 × magnification (Thermo). ImageJ software was then used to measure the mean fluorescence intensity corresponding to each well. Intergroup comparisons were performed on intensity values.

#### 2.7.2. Glycolysis stress assay

Glycolytic activity was assessed *via* extracellular acidification rates (ECARs) [26]. A Seahorse XFe24 Flux Analyzer (Agilent Technologies, Santa Clara, CA, USA.) and a glycolytic stress test kit (Cat. #103020–100; Agilent Technologies, Santa Clara, CA, USA) were used. Briefly, cells were seeded in a Seahorse XF24 Flux Analyzer according to the manufacturer’s protocol and cultured at 37 °C in Seahorse XF DMEM (pH 7.4, Part#: 103575-000, Lot No: 05823001; Agilent Technologies, Santa Clara, CA, USA) overnight. Before the assay, the culture medium was replaced with the assay medium (Seahorse XF Base Medium, pH 7.4, supplemented with 1 mM L-glutamine; Agilent Technologies, Santa Clara, CA, USA), and the cells were subsequently incubated at 37 °C in a non-CO_2_ incubator for 1 h. Following three baseline ECAR measurements, oligomycin (1.0 mM), glucose (10 mM), and 2-deoxy-glucose (50 mM) were added to the cells, and 3 measurements were taken following each injection. The data were analyzed *via* Wave software 2.3.0 (Seahorse Bioscience), and the results were normalized to the cell number/well.

#### 2.7.3. Measurement of lactate levels in supernatants

The lactate level in the VSMCs culture supernatants was determined with a lactic acid assay kit (Nanjing Jiancheng Bioengineering Institute, Nanjing,China,A019-2-1) according to the manufacturer’s protocols.

### 2.8. Pyroptosis assay and detection of IL-1β and LDH in supernatants

For the pyroptotic cell death assays, Hoechst 33342/PI double staining and LDH release assays were performed. Following treatment, VSMCs seeded on coverslips in 6-well plates were treated with a mixture solution of Hoechst 33342 and PI (Beyotime,Shanghai,China, P0137) for 5–10 min. Nuclei were stained with DAPI (Beyotime, Shanghai, China, C1006) for 5 min, and the cells were photographed under a fluorescence microscope.

The secreted IL-1β in VSMCs culture supernatants was detected using a mouse IL-1β ELISA kit (ABclonal,Wuhan, China, RK00006).

Lactate dehydrogenase (LDH) can be released into the extracellular space when cells are damaged or die. To evaluate the proportions of injured and dead cells after the indicated treatments, the concentration of LDH in the culture supernatants was measured. An LDH Kit (Nanjing Jiancheng Bioengineering Institute, Nanjing, China, A020-2-2) was used according to the manufacturer’s instructions.

### 2.9. Transcriptome sequencing analysis

RNA samples were isolated from normally cultured, β-GP-treated, or Irisin + β-GP-cotreated VSMCs. Transcriptome sequencing and analysis were performed by Shanghai OE Biotech Co., Ltd. (Shanghai, China). The annotated DEGs were subjected to Kyoto Encyclopedia of Genes and Genomes (KEGG) enrichment analysis. A volcano plot was drawn *via* a bioinformatics-based approach according to gene expression in different samples (https://www.bioinformatics.com.cn). Significant DEGs were defined as those with an absolute log2 fold change in gene expression value of > 1 and *P* value of < 0.05.

### 2.10. Statistical analysis

All data in this research are presented as the mean ± standard error of the mean (mean ± SEM). GraphPad Prism 6.0 software was used for the statistical analyses (GraphPad Software, La Jolla, CA, USA). One-way analysis of variance (ANOVA) followed by Bonferroni’s post hoc test was performed for comparisons among multiple groups, whereas differences between the two groups were evaluated *via* Student’s *t* test. Each experiment was repeated at least three times. All *p* values <0.05 were considered statistically significant, and *p* values are represented as follows: *p < 0.05, **p < 0.01, ***p < 0.001, ****p < 0.0001.

## 3. Results

### 3.1. Fndc5 (Irisin precursor gene) knockout aggravated aortic calcification in a CKD mouse model

**Figure 1.**
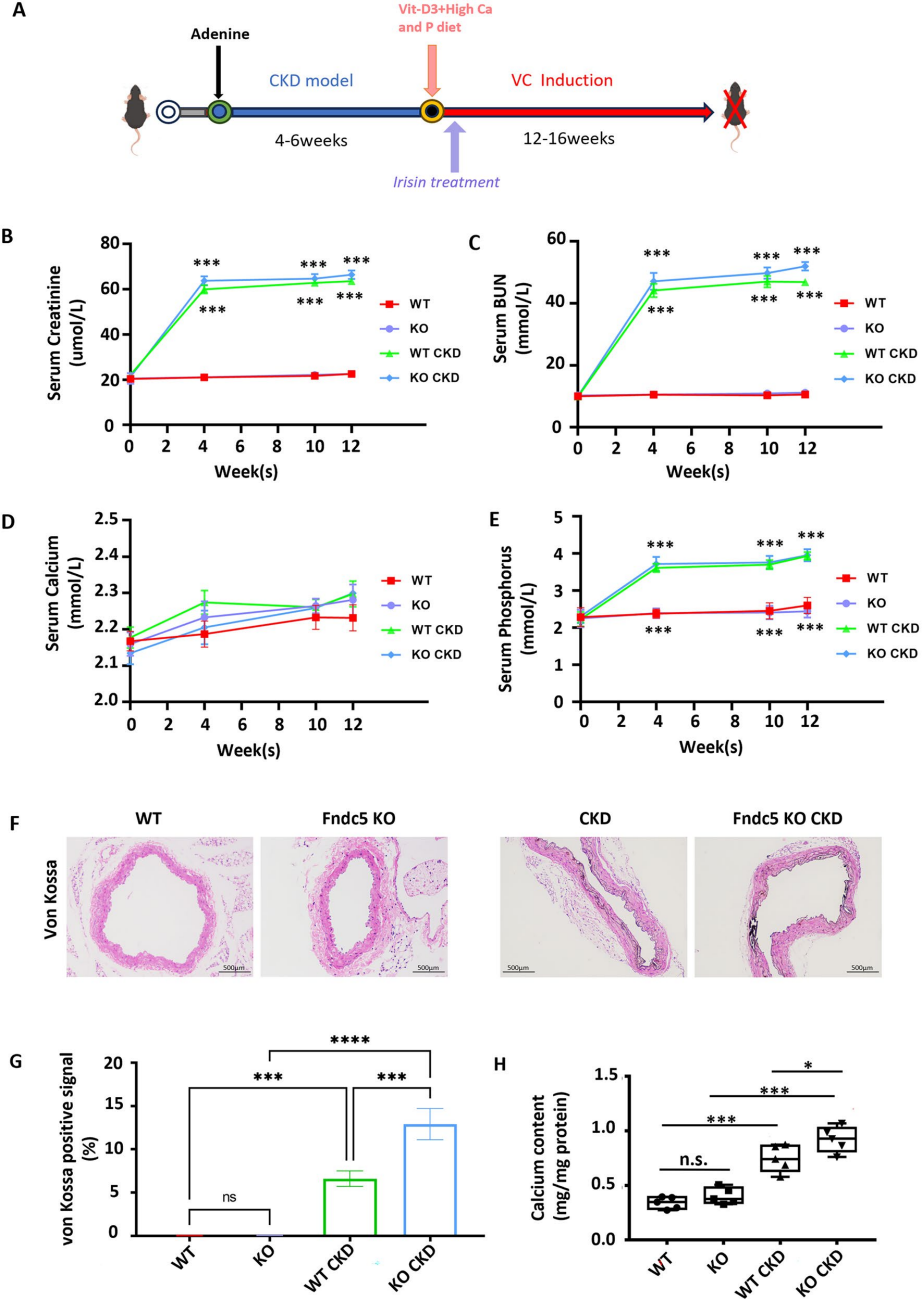
Fndc5 knockout aggravated aortic calcification in a CKD mouse model. (A) Experimental scheme of the CKD-associated VC mouse model. (B–E) Serum levels of creatinine (Cr), urea nitrogen (BUN), calcium (Ca), and phosphate (P) measured. ***p < 0.001, WT CKD compared with the WT group; KO CKD compared with the KO group (*n* = 6). (F) Representative images of Von Kossa-stained aortic rings in different groups (positive staining: black represents calcified tissue, scale bar = 500 μm). (G) Von Kossa positive staining area of mice aortic tissue in different groups. ***p < 0.001, ****p < 0.0001 (H) Calcium content in aortic tissue from each group (*n* = 6). *p < 0.05, **p < 0.01, ***p < 0.001 vs. indicated group; and n.s., not significant. WT: control group; KO: Fndc5 KO group; WT CKD: CKD group; KO CKD: Fndc5 KO CKD group. One-way ANOVA followed by Bonferroni’s hoc test was used for statistical analysis. Results were shown as mean ± SEM.

To determine whether Irisin/Fndc5 is a key factor for CKD-related VC, Fndc5 knockout (Fndc5 KO) CKD mice were constructed. VC was established in CKD model mice according to the experimental scheme (Figure 1(A)). Compared with those in the WT group, the levels of blood Creatinine (Figure 1(B)) and BUN (Figure 1(C)) were significantly increased in the CKD group. Similarly, blood creatinine (Figure 1(B)) and BUN (Figure 1(C)) levels were significantly greater in the Fndc5 KO CKD group than in the Fndc5 KO group. Masson staining revealed glomerulosclerosis and interstitial fibrosis in both the CKD and Fndc5 KO CKD groups (SFig. 1). These results suggest that the CKD model was established successfully. No significant differences in the serum Ca concentration were detected among the 4 groups (Figure 1(D)). The serum P concentration (Figure 1(E)) was significantly greater in the CKD group than in the WT group, and a similar trend was observed in the Fndc5 KO CKD group compared with the Fndc5 KO groups (Figure 1(E)). The mice were subsequently fed a high-calcium and high-phosphorus diet and injected with calcitriol for another 12–16 weeks to induce VC. Von Kossa staining (Figure 1(F)) of the aortic rings revealed no obvious calcium deposition in either the WT or Fndc5 KO mice. However, calcium deposition was significantly greater in the Fndc5 KO CKD group than in the CKD group, as shown by the positive calcification signal of von Kossa staining (Figure 1(G)) and aortic tissue calcium content (Figure 1(H)). Taken together, these data indicate that the deletion of Fndc5 amplified the formation of VC in CKD mice.

### 3.2. Glycolysis increased in β-GP-treated VSMCs, an effect that was inhibited by Irisin

**Figure 2.**
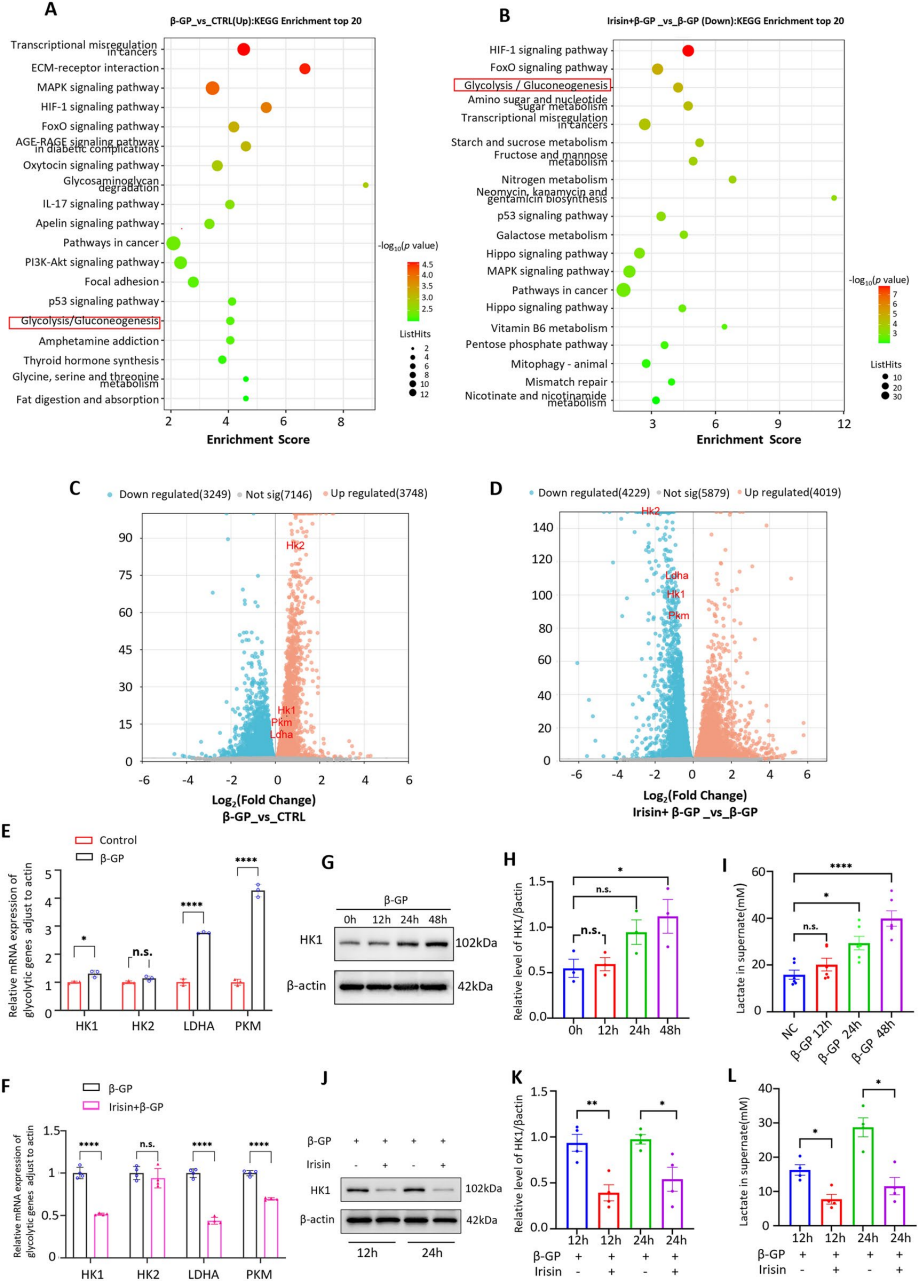
Glycolysis increased in β-GP-treated VSMCs, an effect that was inhibited by Irisin. (A, B) KEGG pathway analysis. The top 20 positively enriched pathways are shown in the bubble chart. (C, D) Volcano plots showing differential gene expression changes. (E, F) Relative mRNA expression of glycolysis-related genes was assessed via RT–qPCR (*n* = 3). *P < 0.05, **P < 0.01, ***P < 0.001, ****P < 0.0001, vs. indicated group; β-GP group: VSMCs were treated with β-GP (10 mM) for 5 days; Irisin + β-GP group: VSMCs were pretreated with Irisin (100 ng/ml) for 24 h before β-GP (10 mM) treatment. CRTL: vehicle control VSMCs. VSMCs were treated with β-GP (10 mM) for the indicated time. (G) HK1 expression in VSMCs was analyzed by Western blot (*n* = 3). (H) Quantification of the results shown in (G). (I) Lactate levels in the culture supernatants were determined by ELISA (*n* = 6). VSMCs were pretreated with or without Irisin (100 ng/ml) in the presence of β-GP (10 mM) for the indicated time. (J) HK1 expression in VSMCs was analyzed by Western blot (*n* = 3). (K) Quantification of the results shown in (J). (L) Lactate levels in the culture supernatants were determined by ELISA (*n* = 6). *P < 0.05, **P < 0.01, ***P < 0.001, ****P < 0.0001, vs. indicated group; and n.s., not significant. One-way ANOVA followed by Bonferroni’s hoc test was used for statistical analysis. Results were shown as mean ± SEM.

To explore the mechanism by which Irisin/Fndc5 inhibit the formation of VC, we performed transcriptome analysis to compare the gene expression among vehicle control VSMCs, VSMCs treated with β-GP, and VSMCs treated with β-GP and Irisin. KEGG enrichment analysis showed that the glycolysis pathway was activated after β-GP treatment (Figure 2(A)). However, the glycolysis pathway was suppressed in the Irisin + β-GP group (Figure 2(B)). Differential expression analysis revealed that the expression of several genes related to glycolysis, including HK1, HK2, LDHA, and PKM, was obviously upregulated after β-GP treatment, whereas the expression of these glycolytic genes was markedly downregulated in the Irisin + β-GP group (Figure 2(C) and (D)). The RT–qPCR results showed that HK1, LDHA, and PKM gene expression presented the highly similar trends to the volcano plot (Figure 2(E, F). Importantly, we found that HK2 gene expression exhibited a nonsignificant trend. Subsequent investigation focused on HK1 as the primary metabolic regulator in this model, as it demonstrated significant and consistent upregulation in response to β-GP, unlike HK2. This is consistent with the established biology wherein HK1 is the predominant isoform responsible for pathological stress responses in differentiated tissues, while HK2 is more frequently associated with proliferative states such as cancer. Western blot analysis also revealed a slight increase in HK1 expression at a very early stage and a further marked increase at 48 h after β-GP treatment (Figure 2(G, H)). In addition to increased HK1 expression, there was a significant increase in the level of lactate, the final product of glycolysis, in the supernatant (Figure 2(I)). Importantly, we found that Irisin inhibited β-GP-induced HK1 expression (Figure 2(J, K)); consistent with this, the lactate level in the culture medium also decreased (Figure 2(L)). These results suggested that HK1-mediated glycolysis was enhanced during VSMCs calcification and that Irisin could counteract these increases in VSMCs under CKD conditions.

### 3.3. Fndc5 (Irisin precursor gene) knockout promoted glycolysis and aggravated calcium deposition in β-GP-treated VSMCs

**Figure 3.**
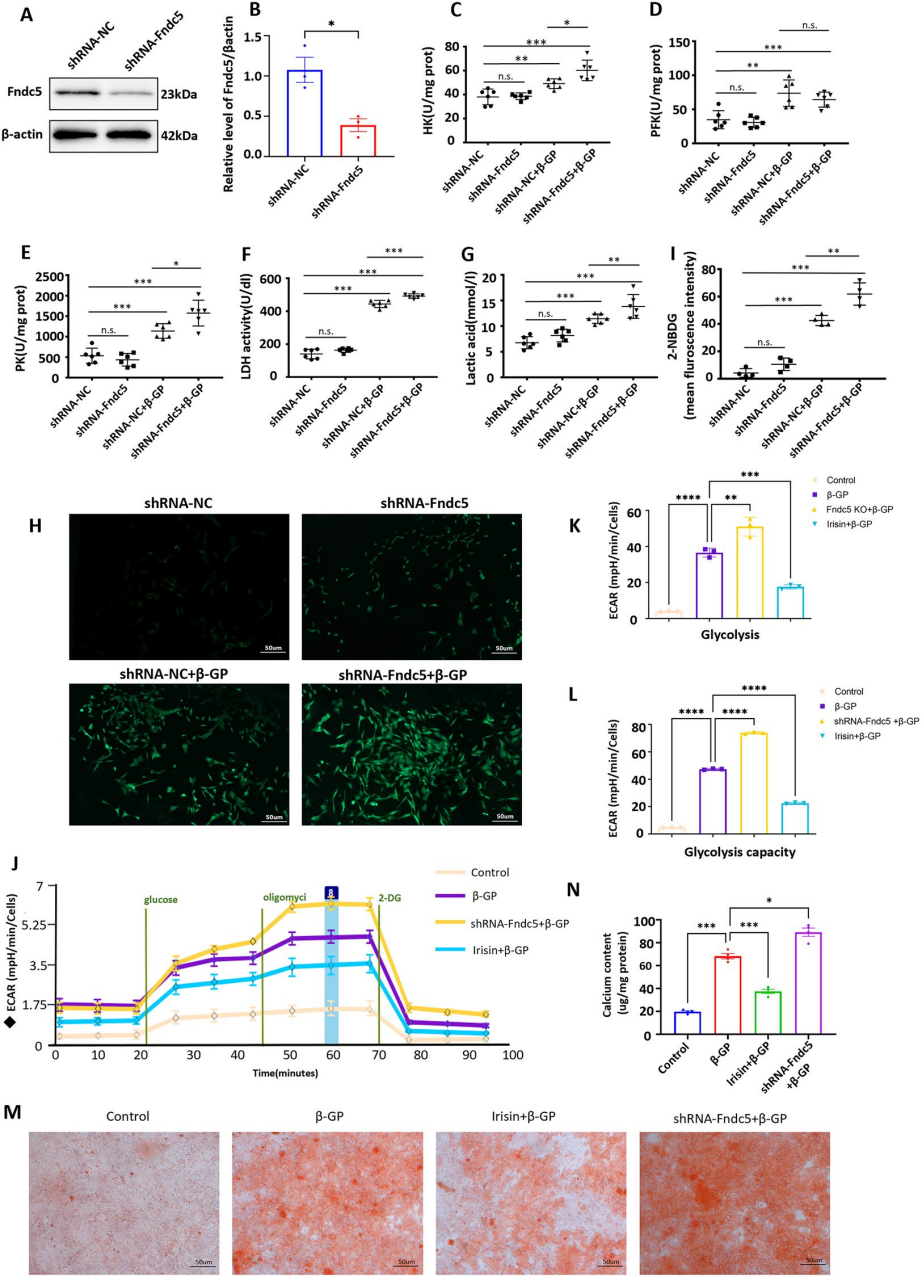
Fndc5 gene knockout promoted glycolysis and aggravated calcium deposition in β-GP-treated VSMCs. (A) Western blot analysis of Fndc5 expression in shRNA-NC VSMCs and shRNA-Fndc5 VSMCs. (B) Quantification of the results shown in (A) (*n* = 3). Student’s *t* test was used for statistical analysis. Results were shown as mean ± SEM. *p < 0.05, vs. shRNA-NC group. (C–N) For shRNA-NC + β-GP group or shRNA-Fndc5 + β-GP group, shRNA-NC VSMCs or shRNA-Fndc5 VSMCs were treated with β-GP (10 mM) for 72 h. In the shRNA-NC + β-GP + Irisin group, shRNA-NC VSMCs were pretreated with Irisin (100 ng/ml) for 24 h before β-GP (10 mM) treatment. (C–F) Activity of glycolysis rate-limiting enzymes (HK, PFK, PK, and LDH) in culture supernatants was determined by ELISA (*n* = 6). (G) Lactate levels in the culture supernatants were determined by ELISA (*n* = 6). (H) Glucose uptake was determined using a 2-NBDG glucose fluorescent probe (*n* = 4). Magnification: ×100; Scale bar = 50 μm; green fluorescence indicates labeled glucose molecules. (I) Quantification of the results shown in (H). (J) Glycolytic activities in VSMCs were measured using a Seahorse glycolysis assay and quantification of ECARs (*n* = 3). (K–L) Glycolysis and glycolysis capacity of the results shown in (J). (M) Calcium deposition in VSMCs was detected by Alizarin red staining (*n* = 3). Positive staining: red represents calcified cells; Scale bar = 100 μm. (N) Quantitative analysis of the calcium content in VSMCs using a Ca assay kit. *p < 0.05, **p < 0.01, **p < 0.01, ***p < 0.001, and ****p < 0.0001, vs. indicated group; and n.s., not significant. One-way ANOVA followed by Bonferroni’s hoc test was used for statistical analysis. Results were shown as mean ± SEM.

To observe the effect of Fndc5 on VSMCs calcification, we cultured shRNA-NC-treated VSMCs and shRNA-Fndc5-treated VSMCs. Figure 3(A, B) shows that Fndc5 protein expression was significantly lower in shRNA-Fndc5 VSMCs than in shRNA-NC VSMCs. The activities of key glycolytic enzymes (HK, PFK, PK, and LDH) and the level of lactate in the culture supernatant were measured to reflect the glycolytic activity. Compared with that in the shRNA-NC group, glycolytic activity was significantly greater in the shRNA-NC + β-GP group. Moreover, glycolytic enzyme (HK, PK, and LDH) activity was greater in the shRNA-Fndc5 + β-GP group than in the shRNA-NC + β-GP group, except PFK (Figure 3(C–G)). Glucose uptake is necessary for glycolysis; we found that the fluorescence intensity of 2-NBDG was significantly greater in the shRNA-NC + β-GP group than in the shRNA-NC group and that the fluorescence intensity of 2-NBDG in the shRNA-Fndc5 + β-GP group was greater than that in the shRNA-NC + β-GP group (Figure 3(H) and (I)). These results revealed that β-GP stimulation increased glucose uptake in VSMCs and that this glucose uptake was further enhanced after Fndc5 knockout. Subsequently, we observed that β-GP stimulation increased the ECAR and increased glycolysis and glycolytic capacity in VSMCs but that Irisin reversed these increases (Figure 3(J, K)). Consistent with the glucose uptake results, Fndc5 knockout increased glycolysis and glycolytic capacity (Figure 3(J–L)). Alizarin red staining revealed that Irisin inhibited β-GP-induced VSMCs calcification, whereas knockout of the Fndc5 gene aggravated β-GP-induced VSMCs calcification (Figure 3(M, N)). These data indicated that the level of glycolysis was elevated in β-GP-induced VSMCs calcification and that the deletion of Fndc5 aggravated the degree of glycolysis and calcium deposition.

### 3.4. HK1 involved in the VSMCs calcification induced by β-GP

**Figure 4.**
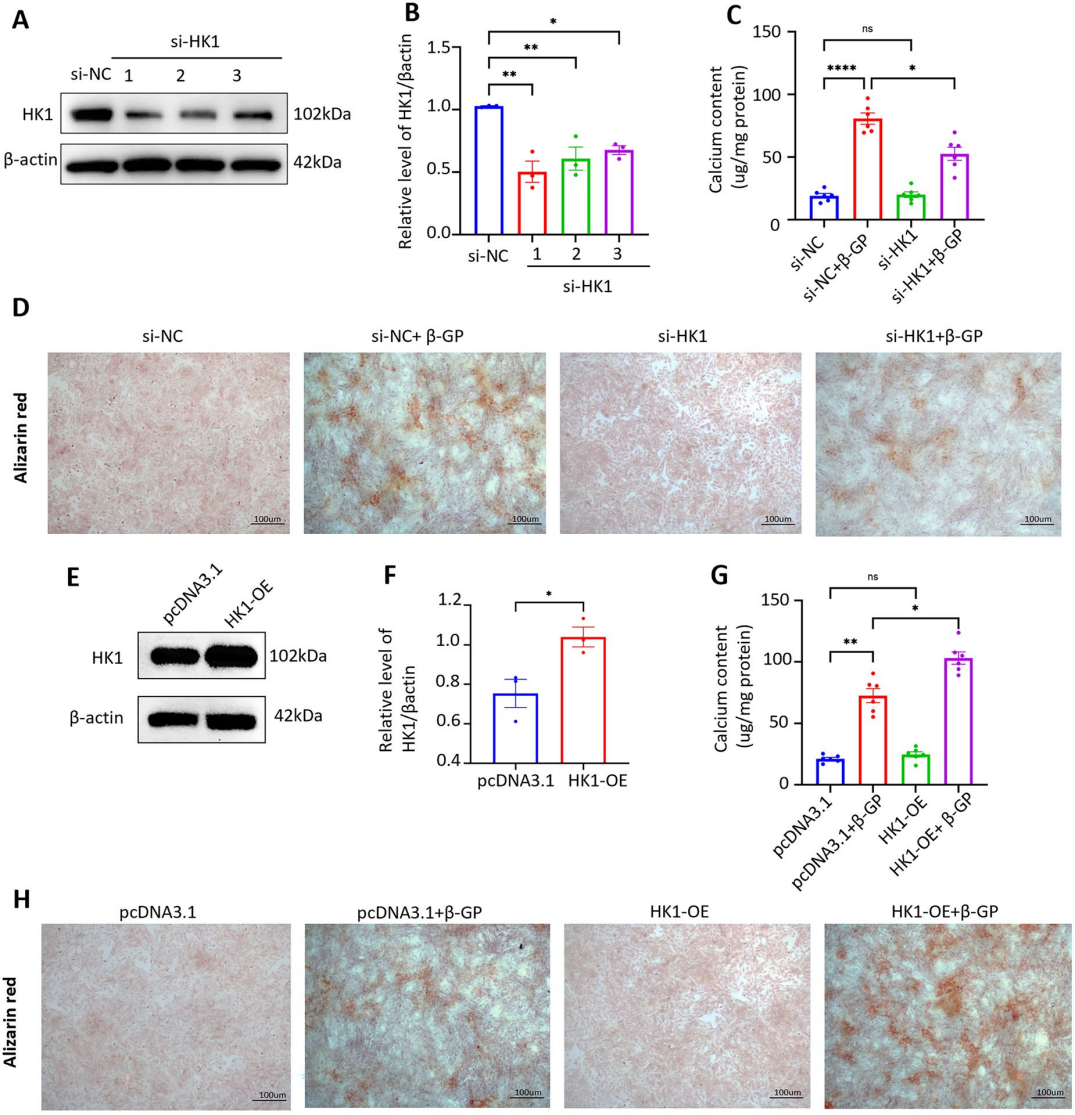
HK1 involved in the VSMCs calcification induced by β-GP. (A) VSMCs were transfected with siRNA against HK1 (si-HK1 1–3) or scrambled sequences (si-NC), knockdown efficiency was measured by Western blot. (B) Representative blots and quantification are shown (*n* = 3). VSMCs transfected with HK1 siRNA were exposed to 10 mM β-GP for 14 days, (C) quantitative analysis of the calcium content in VSMCs using a Ca assay kit., (D) calcium deposition in VSMCs was measured by Alizarin red staining (positive staining: red represents calcified cells; Scale bar = 100 μm) (*n* = 6). VSMCs were transfected with pcDNA 3.1- HK1(HK1-OE) or the pcDNA3.1 empty vector (pcDNA 3.1), (E) Western blot was performed to assess the expression of HK1. (F) Representative blots and quantification are shown in (E) (*n* = 3). VSMCs transfected with HK1-OE were exposed to 10 mM β-GP for 14 days, (G) Quantitative analysis of the calcium content in VSMCs using a Ca assay kit. (H) Calcium deposition in VSMCs was measured by Alizarin red staining (*n* = 6). Positive staining: red represents calcified cells; Scale bar = 100 μm. *P < 0.05, **P < 0.01, ****P < 0.0001, vs. indicated group; n.s., not significant. Student’s *t* test was used for (F). One-way ANOVA followed by Bonferroni’s hoc test was used for (B), (C), and (G). Results were shown as mean ± SEM.

Based on the above results, we next explore the role of HK1 in VSMCs calcification. For the stable knockdown of HK1, three independent siRNAs against mouse HK1 (HK1 1–3) were constructed and transfected into VSMCs, and the results revealed that HK1-1 siRNA significantly inhibited the expression of HK1 in VSMCs. Therefore, we selected the HK1-1 siRNA for subsequent experiments (Figure 4(A, B)). Strikingly, β-GP-induced VSMCs calcification was decreased by HK1 knockdown, as demonstrated by calcium deposition assay (Figure 4(C)) and the Alizarin red staining (Figure 4(D)). In addition, we transfected a plasmid vector carrying the full-length HK1 coding sequence (HK1-OE) into VSMCs. As shown in Figure 4(E) and (F), the HK1 protein expression levels were significantly increased in the VSMCs that were transfected with the HK1-OE plasmids compared with those in the pcDNA3.1 group. Importantly, HK1 overexpression aggravated VSMCs calcification under the high phosphate conditions, as verified by calcium content assays (Figure 4(G)) and Alizarin red staining (Figure 4(H)). These results demonstrated that knockdown of HK1 effectively attenuated calcium deposition in VSMCs induced by β-GP, whereas overexpression of HK1 enhanced the β-GP-induced calcium deposition in VSMCs, indicating that HK1 involved in the process of VSMCs calcification under CKD conditions.

### 3.5. HK1 deficiency attenuated β-GP-induced NLRP3-dependent pyroptotic death in VSMCs

**Figure 5.**
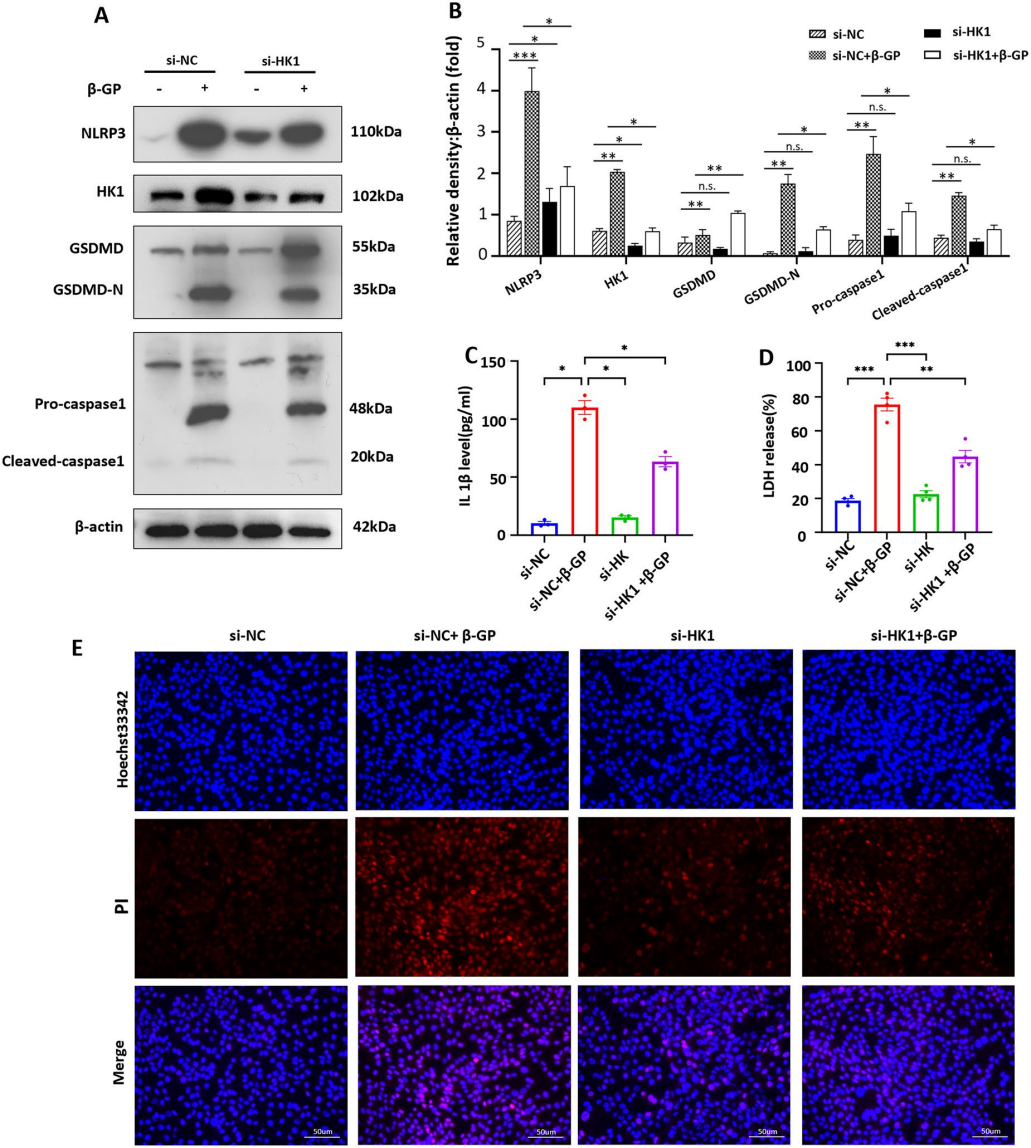
HK1 deficiency attenuated β-GP-induced NLRP3-dependent pyroptotic death in VSMCs. VSMCs were transfected with si-HK1 or scrambled sequence (si-NC) for 48 h and then treated with β-GP (10 mM) for 5 days. (A) Western blot was performed to assess the expression of NLRP3, HK1, GSDMD-N, and Caspase-1. (B) Quantification of the results shown in (A) (*n* = 3). (C) IL-1β proteins levels in the cell culture media by ELISA (*n* = 3). (D) LDH release was detected *via* an LDH assay kit (*n* = 4). (E) Double-fluorescence staining with Hoechst 33342 (blue) and PI (red) (*n* = 3). Scale bar = 50 μm. *P < 0.05, **P < 0.01, ***P < 0.001, vs indicated group; n.s., not significant. One-way ANOVA followed by Bonferroni’s hoc test was used for statistical analysis. Results were shown as mean ± SEM.

Our previous study demonstrated that NLRP3-dependent pyroptotic cell death is a critical mechanism of VSMCs calcification [21]. Recent research has revealed that glycolysis is an important upstream regulator of NLRP3 inflammasome activation. Therefore, we aimed to determine the relationship between HK1- and NLRP3-dependent pyroptosis during VSMCs calcification. Figure 5(A) and (B) shows that β-GP significantly increased the protein expression of NLRP3 and pyroptosis-related markers (activated caspase-1, GSDMD-N) in VSMCs. Moreover, the IL-1β production (Figure 5(C)), LDH release (Figure 5(D)) and the number of PI-positive cells (Figure 5(E)) also markedly increased in the β-GP group, suggesting that the plasma membrane ruptured and became leaky during VSMCs calcification. These results demonstrated that pyroptotic cell death occurred in β-GP treated VSMCs, consistent with our previous study [21]. Subsequently, we found that the β-GP-induced increase in the protein expression of NLRP3 in VSMCs was significantly reduced by HK1 knockdown (Figure 5(A, B)). Importantly, inhibition of HK1 also decreased the protein levels of pyroptosis-related markers (Figure 5(A, B)), IL-1β secretion (Figure 5(C)), LDH release (Figure 5(D)), and the number of PI-positive cells (Figure 5(E)) in β-GP-treated VSMCs. These findings revealed the critical role of HK1 in β-GP-induced NLRP3 inflammasome activation and pyroptotic cell death in VSMCs.

### 3.6. HK1 overexpression abolished the inhibitory effect of Irisin on β-GP-induced VSMCs pyroptosis and calcification

**Figure 6.**
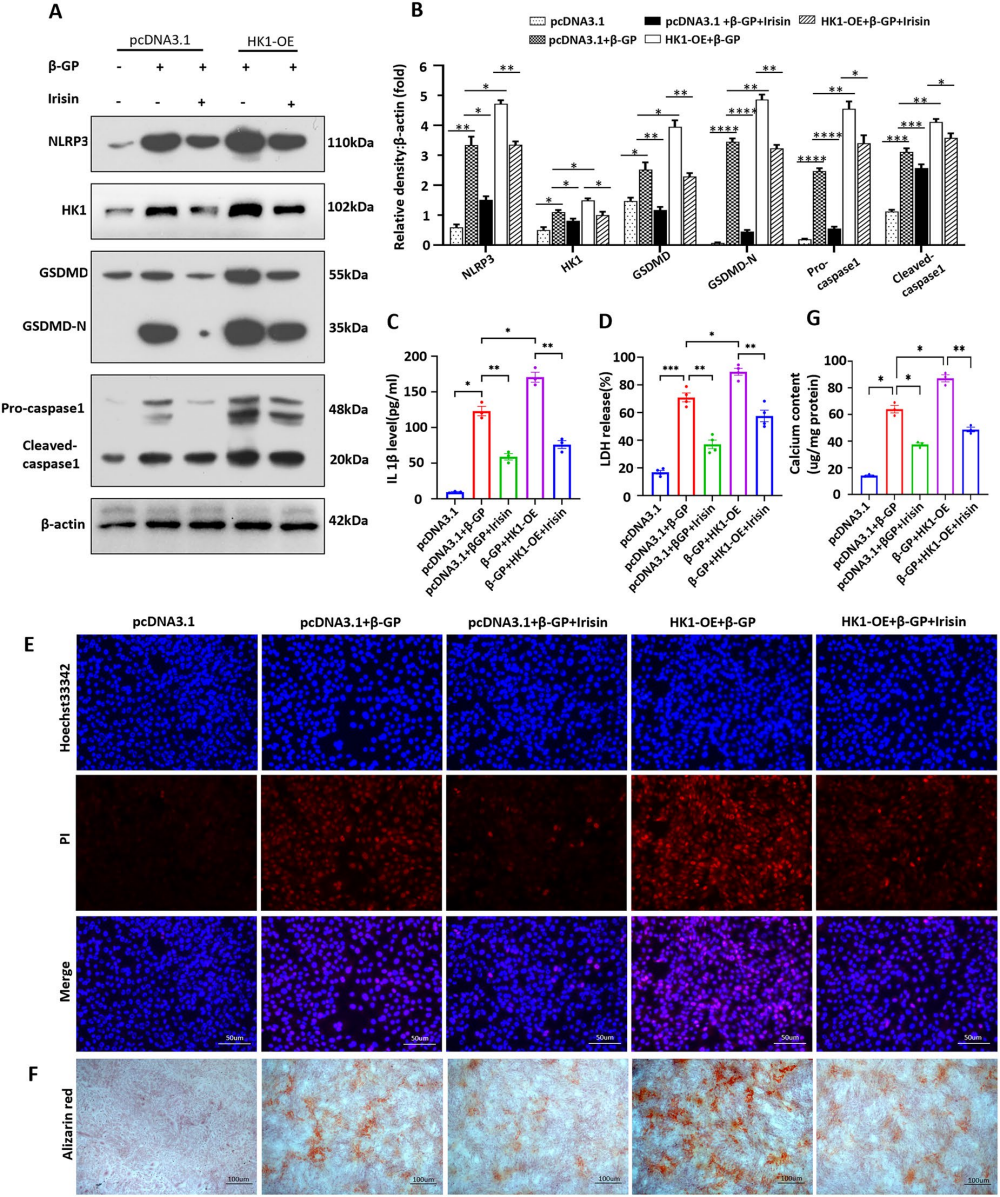
HK1 overexpression abolished the inhibitory effect of Irisin on β-GP-induced VSMC pyroptosis and calcification. VSMCs were transfected with either a pcDNA3.1 empty vector (pcDNA3.1) or HK1-overexpression plasmid (HK1-OE), and subsequently treated with β-GP (10 mM) with or without Irisin (100 ng/ml) for 5 days. (A) Western blot was performed to assess the expression of NLRP3, HK1, GSDMD-N, and Caspase-1. (B) Quantification of the results shown in (A) (*n* = 3). (C) IL-1β proteins levels in the cell culture media by ELISA (*n* = 3). (D) LDH release was detected *via* an LDH assay kit (*n* = 4). (E) Double-fluorescence staining with Hoechst 33342 (blue) and PI (red). Scale bar = 50 μm. (F) Calcium deposition in VSMCs was measured by Alizarin red staining. (G) Quantitative analysis of the calcium content in VSMCs using a Ca assay kit (*n* = 3). Positive staining: red represents calcified cells; Scale bar =100 μm. *P < 0.05, **P < 0.01, ***P < 0.001, ****P < 0.0001, vs indicated group; n.s., not significant. One-way ANOVA followed by Bonferroni’s hoc test was used for statistical analysis. Results were shown as mean ± SEM.

To further explore the mechanism by which Irisin inhibits VSMCs pyroptosis and calcification, we transfected an HK1 overexpression plasmid (HK1-OE) into VSMCs and treated them with β-GP in the presence or absence of Irisin. The results showed that β-GP treatment markedly increased the protein expression levels of HK1, NLRP3 and pyroptosis-related markers (Figure 6(A, B)) and increased IL-1β secretion (Figure 6(C)), LDH release (Figure 6(D)), and the number of PI-positive VSMCs (Figure 6(E)). As expected, HK1 overexpression significantly aggravated NLRP3-dependent pyroptotic cell death in β-GP-induced VSMCs (Figure 6(A–E)). Importantly, Irisin markedly inhibited the β-GP-induced expression of HK1, which was accompanied by reduced protein expression of NLRP3 and pyroptosis-related markers (Figure 6(A, B)). Consistent with the changes in protein levels, the level of released LDH (Figure 6(D)), IL-1β secretion (Figure 6(C)), and the number of PI-positive cells (Figure 6(E)) showed similar trends, suggesting that Irisin inhibited β-GP-induced pyroptotic cell death in VSMCs, whereas the inhibitory effect of Irisin on NLRP3-dependent pyroptotic cell death was partly abolished when HK1 was overexpressed (Figure 6(B–E)). In addition, as shown in Figure 6(F) and (G), β-GP treatment markedly increased calcium deposition in VSMCs, and Irisin treatment inhibited this phenomenon. Most importantly, overexpression of HK1 significantly attenuated the inhibitory effect of Irisin on calcium deposition in VSMCs. Together, our results verified that the overexpression of HK1 reversed the protective effect of Irisin against β-GP-induced pyroptosis and calcification in VSMCs.

### 3.7. Irisin/Fndc5 deficiency promoted the VSMCs glycolysis and pyroptosis and accelerated medial calcification in CKD mice

**Figure 7.**
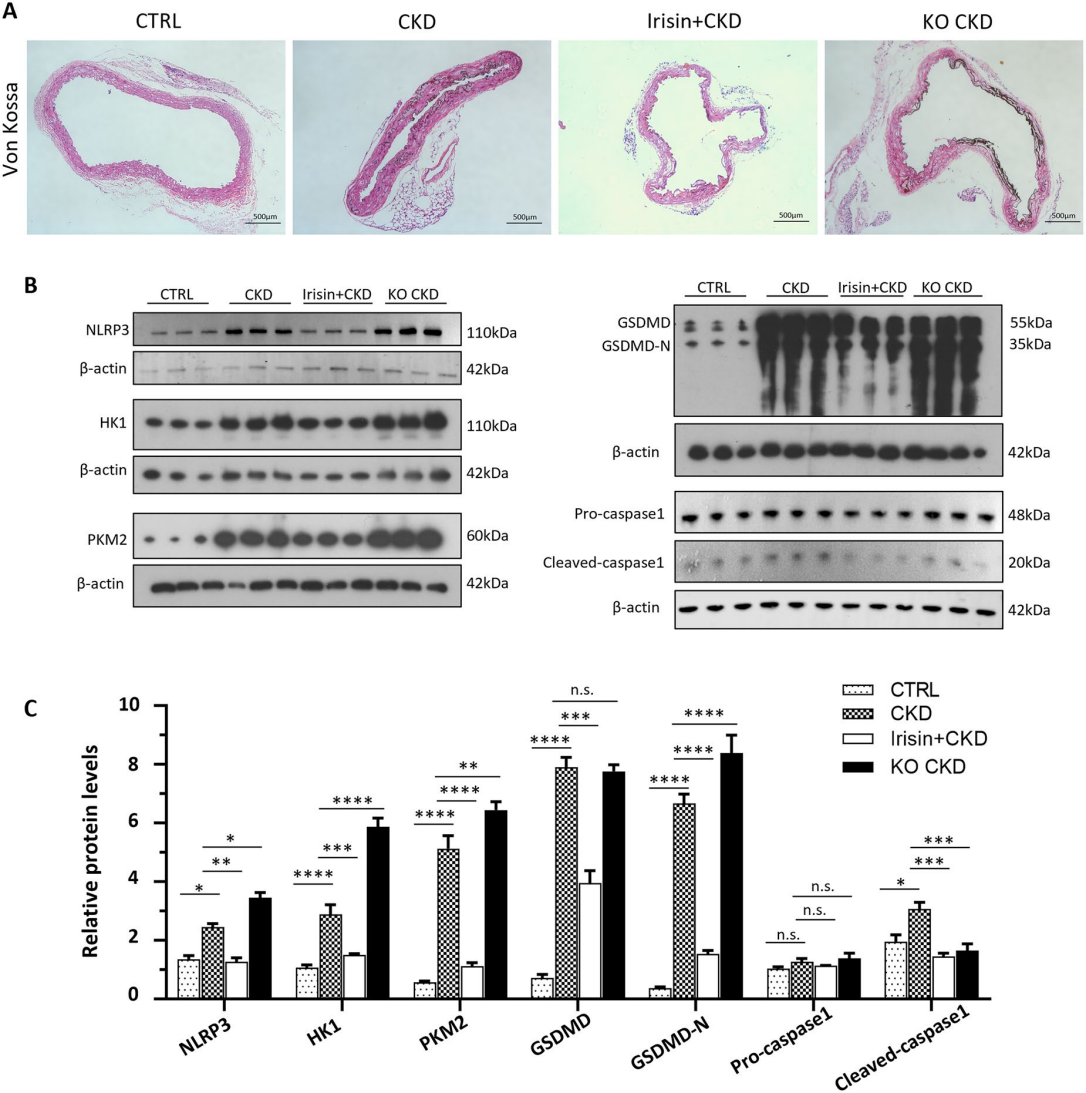
Irisin/FNDC5 deficiency promoted VSMC glycolysis and pyroptosis and accelerated medial calcification in CKD mice. (A) Representative images of Von Kossa-stained aortas from various groups of mice (*n* = 6). Scale bar = 500 μm. (B) The protein levels of HK1, PKM2, and pyroptosis-related markers in the aortas of the different groups of mice were assessed by Western blot (*n* = 6). (C) Quantification of the results shown in (B). *P < 0.05, **P < 0.01, ***P < 0.001, ****P < 0.0001 vs. indicated group; n.s., not significant. CTRL: WT mice; CKD: CKD mice; Irisin + CKD: CKD mice treated with Irisin; KO CKD: Fndc5 KO CKD mice. One-way ANOVA followed by Bonferroni’s hoc test was used for statistical analysis. Results were shown as mean ± SEM.

*In vivo* study revealed that Irisin treatment effectively reduced aortic calcification in CKD mice and that there was more severe calcification in the Fndc5-KO CKD group than in the CKD group (Figure 7(A)). Western blot results revealed increased expression of key glycolytic enzymes (HK1 and PKM) and pyroptosis-related proteins (NLRP3, GSDMD-N, and Cleaved-caspase-1) in the calcified aortas of the mice in the CKD group compared with those in the aortas of the mice in the control group. Irisin treatment not only decreased the expression of key glycolytic enzymes but also decreased the expression of pyroptosis-related proteins (Figure 7(B)). The abovementioned indicators were also higher in the Fndc5 KO CKD group than in the CKD group except Cleaved-caspase-1, as shown by Western blot (Figure 7(B)). The comparatively lower level of cleaved caspase-1 in the Fndc5 KO CKD group, despite the increase in upstream (NLRP3) and downstream (GSDMD-N) markers, may reflect the transient nature of caspase-1 activation during pyroptosis. At the stage represented by this group, the initial wave of caspase-1 cleavage may have subsided, leaving behind the more stable GSDMD-N fragments as a marker of executed pyroptosis. Taken together, these results demonstrated that Irisin treatment inhibited VSMC glycolysis and thereby suppressed pyroptosis and medial calcification in the aortic tissues of CKD mice, whereas Fndc5 knockout promoted VSMCs glycolysis and pyroptotic cell death and exacerbated aortic medial calcification in CKD mice.

## 4. Discussion

In this study, we discovered that HK1-mediated glycolysis promoted VC in CKD by accelerating NLRP3 inflammasome activation and subsequent VSMCs pyroptosis, highlighting a novel role for HK1-mediated glycolysis in CKD-associated VC formation. Moreover, we found that Irisin plays a protective role in VC *via* the suppression of HK1-mediated glycolysis and subsequent VSMCs pyroptosis during CKD. Importantly, knockout of the Irisin precursor Fndc5 deteriorated the formation of VC in CKD by promoting glycolysis and pyroptosis in VSMCs.

Glycolysis is a metabolic pathway that converts glucose into pyruvate, playing a crucial role in energy production and generating metabolites that are essential for cell function [27]. Recent studies have highlighted the significant role of glycolysis in cardiovascular diseases such as atherosclerosis [28], myocardial ischemia–reperfusion [29], and cardiac hypertrophy [30], revealing complex interactions between metabolic pathways and cardiovascular health. However, the possible mechanism by which glycolysis promotes VC remains unclear. In this study, we observed enhanced glycolytic activity during VSMCs calcification, represented by increased glucose uptake, the activity of glycolysis rate-limiting enzymes, lactate production, and the ECAR during VSMCs calcification, revealing that glycolysis might be involved in VSMCs calcification. Transcriptome analysis and differential expression analysis revealed that the key glycolysis gene of HK1 was obviously elevated during VSMCs calcification, and that genetic enhancement of HK1 exacerbated VSMCs glycolytic activity and calcification, whereas HK1 deficiency ameliorated these phenotypes. Our findings are consistent with results reported by Liu [2] but not with the results reported by He and Ioana Alesutan [7]. One possible explanation for this discrepancy is that the increase in glycolysis is a passive process resulting from the mitochondrial dysfunction induced by β-GP [7,31]; therefore, it would be difficult to observe the elevated glycolysis levels if the period of high phosphorus stimulation was too short. Therefore, our results suggest that HK1-mediated glycolysis is an important mechanism involved in CKD-associated VC.

Pyroptosis, which shares characteristics with both apoptosis and necrosis, is mediated by NLRP3 inflammasome activation. Accumulating evidence has demonstrated that NLRP3 inflammasome activation-mediated pyroptosis is involved in many cardiovascular diseases [32–34]. As we reported previously [21], vascular calcification in CKD is triggered by NLRP3 inflammasome-mediated pyroptosis in VSMCs. This process is initiated by β-GP, which induces the ROS generation in VSMCs, thereby activating the NLRP3 inflammasome and subsequently leading to pyroptosis. Moreover, recent studies have shown that glycolysis is an important trigger for the activation of the NLRP3 inflammasome. Liu et al. demonstrated that PKM2-dependent glycolysis promotes skeletal muscle cell pyroptosis by activating the NLRP3 inflammasome in dermatomyositis/polymyositis [35]. Zhong et al. revealed that the glycolytic enzyme PGK1 promotes M1 macrophage polarization and induces pyroptosis in acute lung injury through the regulation of NLRP3 [36]. Thus, we speculated that HK1-mediated glycolysis is involved in the CKD-associated VC through the promotion of NLRP3 inflammasome activation and VSMCs pyroptosis. In this study, we found that HK1 overexpression promoted glycolysis-related gene expression and lactate levels in β-GP treated VSMCs, and further accelerated NLRP3-dependent VSMCs pyroptosis and calcification. Importantly, β-GP-induced NLRP3 inflammasome activation and VSMCs pyroptosis were reversed by the inhibition of HK1 expression. Together, our results reveal a new mechanism of the critical role of glycolysis in VSMCs calcification: HK1-mediated glycolysis promotes VSMCs calcification by accelerating NLRP3 inflammasome activation and pyroptotic cell death.

Irisin is a newly discovered myokine that is generated by the cleavage of its precursor Fndc5 in response to exercise [37]. Increasing evidence has revealed that Irisin is present in almost all human organs, acts as a gatekeeper of metabolic energy regulation, and plays an important role in maintaining glucose and lipid homeostasis and combating various diseases [38–40]. Peng et al. provided evidence that Irisin administration can stimulate aerobic metabolism of both glucose and fatty acids in kidney tubule cells, and improve kidney function and attenuate kidney fibrosis in mouse models of CKD [41]. A lower serum Irisin level has been reported in patients with diabetes, coronary artery disease and uremic disease [42–44]. Our earlier study [31] shown that Irisin reverses Runx2 and BMP2 overexpression, hence suppressing β-GP-induced osteoblastic transformation in VSMCs through the AMPK/Drp1 signaling pathway. In our current study, we observed that the increased HK1 protein and lactate levels in β-GP-induced VSMCs were reversed by Irisin treatment. Most importantly, knockout of the Irisin precursor Fndc5 accelerated the β-GP-induced glycolysis and calcification in VSMCs, and Fndc5 deficiency aggravated the aortic calcification in CKD mice. These results indicate that Irisin/Fndc5 plays an important protective role against VC by regulating VSMCs glycolysis and pyroptosis in CKD.

Some limitations in our study must be noted. First, the absence of *in vivo* validation data for glycolytic flux changes, particularly measurements of lactate and inflammasome markers (IL-1β/IL-18), constitutes a study limitation. Second, further studies are needed to explore the regulatory mechanism of Irisin on the downstream HK1-mediated glycolytic pathway in the development of VC in CKD. Third, the lack of clinical correlation between serum Irisin levels and vascular calcification severity in CKD patients is a recognized limitation, and we agree that such studies constitute a valuable future endeavor.

## 5. Conclusion

This study demonstrates for the first time that HK1-driven glycolysis functions as a proximal metabolic trigger of NLRP3 inflammasome activation and pyroptosis in CKD-associated vascular calcification. Under the high-phosphate conditions, HK1 upregulation enhances glycolytic flux, promoting NLRP3-dependent pyroptotic death of VSMCs and accelerating calcium deposition. Irisin/FNDC5 counteracts this pathological process by suppressing HK1 expression and glycolytic activation, thereby attenuating inflammasome assembly and pyroptosis. Genetic deletion of Fndc5 exacerbated HK1–NLRP3 signaling and medial calcification in CKD mice, whereas enforced HK1 expression abolished the protective effect of Irisin. Collectively, these findings identify the HK1–glycolysis–NLRP3 axis as a critical metabolic–inflammatory link in CKD-related vascular calcification and suggest that Irisin/HK1 targeting may represent a promising therapeutic strategy to disrupt pyroptosis-driven vascular injury in CKD. Future studies are warranted to explore pharmacologic modulation of HK1 or Irisin analogs as metabolic–immunologic interventions for vascular calcification in CKD.

## Supplementary Material

ARRIVE Guidelines Checklist.docx

## Data Availability

The data used to support the findings of this study are available upon request from the corresponding author.
