# Supplementary material for: Irisin targets the HK1–glycolysis–NLRP3 pyroptosis axis to prevent chronic kidney disease-associated vascular calcification
Source: Ren Fail. 2026 Jan 7;47(1):2610545. doi: 10.1080/0886022X.2025.2610545 (PMC12784635; doi:10.1080/0886022X.2025.2610545)
Supplement: ARRIVE Guidelines Checklist.docx [file IRNF_A_2610545_SM2258.docx]

**The ARRIVE Essential 10**

These items are the basic minimum to include in a manuscript. Without this information, readers and reviewers cannot assess the reliability of the findings.

| Item | Recommendation | Section/line number, or reason for not reporting |
| --- | --- | --- |
| **Study design** | 1 For each experiment, provide brief details of study design including: a. The groups being compared, including control groups. If no control group has been used, the rationale should be stated. b. The experimental unit (e.g. a single animal, litter, or cage of animals). | **a & b:** Figure 1A and **Section 2.1 (Lines 97-120)** describe the in vivo experimental groups (WT, WT CKD, Irisin+CKD, Fndc5 KO, Fndc5 KO CKD) and the use of control groups. The experimental unit is a single mouse. **Section 2.2 (Lines 128-144)** describes in vitro experimental groups and treatments (e.g., control, β-GP, β-GP+Irisin, shRNA-NC, shRNA-Fndc5). The experimental unit is a single culture well/dish. |
| **Sample size** | 2 a. Specify the exact number of experimental units allocated to each group, and the total number in each experiment. Also indicate the total number of animals used. b. Explain how the sample size was decided. Provide details of any a priori sample size calculation, if done. | **a:** The exact sample size (n) for each experiment is provided in the **figure legends** (e.g., Lines 664-672 for Fig 1: n=6; Fig 2: n=3-6; Fig 3: n=3-6; etc.). **b:** The sample size was determined based on literature reports and statistical analysis. |
| **Inclusion and exclusion criteria** | 3 a. Describe any criteria used for including and excluding animals (or experimental units) during the experiment, and data points during the analysis. Specify if these criteria were established a priori. If no criteria were set, state this explicitly. b. For each experimental group, report any animals, experimental units or data points not included in the analysis and explain why. If there were no exclusions, state so. c. For each analysis, report the exact value of n in each experimental group. | **a:** The experimental animals were selected for each group according to the inclusion requirements, and those whose gender and weeks of age did not meet the inclusion requirements, whose general condition was poor after intervention were excluded. The inclusion criteria for experimental animals were described in **Section 2.1(Lines 101).**  **b:** In each experimental group, the experimental animals whose general situation was poor, and renal function did not meet the requirements were not included in the data analysis, because data consistency would be affected.  **c:** The exact sample size (n) for each experiment is provided in the **figure legends.** |
| **Randomisation** | 4 a. State whether randomisation was used to allocate experimental units to control and treatment groups. If done, provide the method used to generate the randomisation sequence. b. Describe the strategy used to minimise potential confounders such as the order of treatments and measurements, or animal/cage location. If confounders were not controlled, state this explicitly. | **a:** The experimental animals were randomly grouped described in **Section 2.1(Lines 107-109).** The method of randomisation is not specified. No information is provided on the control of potential confounders.  **b:** To reduce potential disturbance, laboratory animals were housed in standard animal rooms, and kept in controlled environment. Methods of experimental animals in each group were clearly described in **Section 2.1(Line 98-126).** |
| **Blinding** | 5 Describe who was aware of the group allocation at the different stages of the experiment (during the allocation, the conduct of the experiment, the outcome assessment, and the data analysis). | Under the guidance of corresponding author, the first two authors assisted to complete the experiment, including experiment allocation, experiment implementation, experiment result evaluation and data analysis, while the fourth and fifth authors assisted in data analysis and article writing. |
| **Outcome measures** | 6 a. Clearly define all outcome measures assessed (e.g. cell death, molecular markers, or behavioural changes). b. For hypothesis-testing studies, specify the primary outcome measure, i.e. the outcome measure that was used to determine the sample size. | **a:** Outcome measures are clearly defined in the Methods: calcification (Alizarin Red, Von Kossa, calcium content, **Lines 146-162**), protein levels (Western Blot, **Lines 164-178**), gene expression (RT-qPCR, **Lines 180-188**), glycolysis (enzyme activity, lactate, glucose uptake, ECAR, **Lines 198-225**), pyroptosis (IL-1β, LDH, PI staining, **Lines 227-238**). **b:** No applicable. Our study was not a hypothesis testing study. |
| **Statistical methods** | 7 a. Provide details of the statistical methods used for each analysis, including software used. b. Describe any methods used to assess whether the data met the assumptions of the statistical approach, and what was done if the assumptions were not met. | **a:** The statistical methods and software for  statistical methods were described **in Section 2.10 (Line 248-254)**.  **b:** The data were analyzed and evaluated by statisticians. |
| **Experimental animals** | 8 a. Provide species-appropriate details of the animals used, including species, strain and substrain, sex, age or developmental stage, and, if relevant, weight. b. Provide further relevant information on the provenance of animals, health/immune status, genetic modification status, genotype, and any previous procedures. | **a:** Detailed information on experimental animals were described in **Section 2.1(Lines 101-106)**.  **b:** The provenance of animals was National Model Animal Research Center of Nanjing University. Fndc5-KO C57BL/6J mice (Fndc5 KO mice) were purchased from Jiangsu Gem Pharmatech Biological Company, described in **Section 2.1(Lines 101-105)**.  . |
| **Experimental procedures** | 9 For each experimental group, including controls, describe the procedures in enough detail to allow others to replicate them, including: a. What was done, how it was done and what was used. b. When and how often. c. Where (including detail of any acclimatisation periods). d. Why (provide rationale for procedures). | **a, b, c, d:** Detailed procedures for in vivo CKD and calcification models **(Lines 110-120)**, cell culture and treatments **(Lines 128-144)**, and all experimental assessments **(Lines 146-246)** are provided, including durations, concentrations, and sources of reagents. |
| **Results** | 10 For each experiment conducted, including independent replications, report: a. Summary/descriptive statistics for each experimental group, with a measure of variability where applicable (e.g. mean and SD, or median and range). b. If applicable, the effect size with a confidence interval. | **a:** All figures show summary data (mean) with error bars (SEM) for each group. The figure legends state the number of independent replicates (n). **b:** No applicable. Our study strictly followed the ARRIVE guidelines and minimized the impact on experimental results. |

**The Recommended Set**

These items complement the Essential 10 and add important context to the study. Reporting the items in both sets represents best practice.

| Item | Recommendation | Section/line number, or reason for not reporting |
| --- | --- | --- |
| **Abstract** | 11 Provide an accurate summary of the research objectives, animal species, strain and sex, key methods, principal findings, and study conclusions. | We had described the research objectives, main methods and findings, and conclusions in Section **Abstract (Lines 23-42)**. |
| **Background** | 12 a. Include sufficient scientific background to understand the rationale and context for the study, and explain the experimental approach. b. Explain how the animal species and model used address the scientific objectives and, where appropriate, the relevance to human biology. | **a:** The background of this research had been explained in **Section Introduction (Lines 47-94)** provide extensive background on VC, CKD, glycolysis, Irisin, and pyroptosis, establishing the rationale. **b:** The use of mouse VSMCs and a well-established mouse model of CKD-mediated vascular calcification **(Lines 110-120)** is standard for investigating this human pathology. The relevance to human biology is discussed in the **Introduction and Discussion**. |
| **Objectives** | 13 Clearly describe the research question, research objectives and, where appropriate, specific hypotheses being tested. | Research questions and objectives were clearly described in **Section Introduction (Lines 88-94).** |
| **Ethical statement** | 14 Provide the name of the ethical review committee or equivalent that has approved the use of animals in this study, and any relevant licence or protocol numbers (if applicable). If ethical approval was not sought or granted, provide a justification. | The Ethical statement was described in **Section2.1 (Lines 98-101) and Ethical approval (Lines 512-513)**: Institutional Animal Care and Use Committee (IACUC) of Capital Medical University and The Lab Animal Ethical Committee of Capital Medical University. |
| **Housing and husbandry** | 15 Provide details of housing and husbandry conditions, including any environmental enrichment. | Laboratory animals were housed in a standard animal room, and kept in controlled environment, described in **Section 2.1(Line 98-126).** |
| **nimal care and monitoring** | 16 a. Describe any interventions or steps taken in the experimental protocols to reduce pain, suffering and distress. b. Report any expected or unexpected adverse events. c. Describe the humane endpoints established for the study, the signs that were monitored and the frequency of monitoring. If the study did not have humane endpoints, state this. | **a:** Anaesthesia (isoflurane) for procedures and euthanasia is mentioned **(Line 121)**. **b:** No adverse events. Our study strictly followed the ARRIVE guidelines, and fully concerned about the safety of laboratory animals.  **c:** Specific humane endpoints are not described. The successful establishment of the CKD model was confirmed by measuring BUN and Creatinine **(Lines 113-114)**, which are indicators of animal health. |
| **Interpretation/ scientific implications** | 17 a. Interpret the results, taking into account the study objectives and hypotheses, current theory and other relevant studies in the literature. b. Comment on the study limitations including potential sources of bias, limitations of the animal model, and imprecision associated with the results. | **a:** The **Discussion section** **(Lines 410-484)** thoroughly interprets the results in the context of the objectives and existing literature. **b:** Study limitations are explicitly discussed in **Lines 477-484**. |
| **Generalisability/ translation** | 18 Comment on whether, and how, the findings of this study are likely to generalise to other species or experimental conditions, including any relevance to human biology (where appropriate). | The relevance to human biology (CKD patients) is a central theme of the paper, mentioned in the Introduction (Lines 47-53) and Conclusion (Lines 487-501). The mechanistic link (HK1-glycolysis-NLRP3) suggests potential generalizability. But this study is a basic study, whether the results can be applied to other species or experimental conditions requires further validation. Anthropological research requires ethics committee approval. |
| **Protocol registration** | 19 Provide a statement indicating whether a protocol (including the research question, key design features, and analysis plan) was prepared before the study, and if and where this protocol was registered. | A protocol was prepared before the study  without registration. |
| **Data access** | 20 Provide a statement describing if and where study data are available. | **Lines 526-527**: "The data used to support the findings of this study are available upon request from the corresponding author." |
| **Declaration of interests** | 21 a. Declare any potential conflicts of interest, including financial and non-financial. If none exist, this should be stated. b. List all funding sources (including grant identifier) and the role of the funder(s) in the design, analysis and reporting of the study. | **a:** The authors have no competing interests to declare **Section Disclosure statement (Line 517).** **b:** **Section Funding (Lines 520-523)** list all funding sources and grant numbers. The role of the funders is not specified. |
